# Driver mutation zygosity is a critical factor in predicting clonal hematopoiesis transformation risk

**DOI:** 10.1038/s41408-023-00974-9

**Published:** 2024-01-15

**Authors:** Ashwin Kishtagari, M. A. Wasay Khan, Yajing Li, Caitlyn Vlasschaert, Naimisha Marneni, Alexander J. Silver, Kelly von Beck, Travis Spaulding, Shannon Stockton, Christina Snider, Andrew Sochacki, Dixon Dorand, Taralynn M. Mack, P. Brent Ferrell, Yaomin Xu, Cosmin A. Bejan, Michael R. Savona, Alexander G. Bick

**Affiliations:** 1 Division of Hematology/Oncology, Vanderbilt University School of Medicine, Nashville, TN USA; 2 Vanderbilt-Ingram Cancer Center, Vanderbilt University School of Medicine, Nashville, TN USA; 3 Division of Genetic Medicine, Vanderbilt University Medical Center, Nashville, TN USA; 4 Department of Biostatistics, Vanderbilt University School of Medicine, Nashville, TN USA; 5 Department of Medicine, Queen’s University, Kingston, ON Canada; 6 Program in Cancer Biology, Vanderbilt University School of Medicine, Nashville, TN USA; 7 Vanderbilt University School of Medicine, Nashville, TN USA; 8 Department of Medicine, Vanderbilt University Medical Center, Nashville, TN USA; 9 Center for Quantitative Sciences, Vanderbilt University School of Medicine, Nashville, TN USA; 10 Department of Biomedical Informatics, Vanderbilt University School of Medicine, Nashville, TN USA; 11 Center for Immunobiology, Vanderbilt University School of Medicine, Nashville, TN USA

**Keywords:** Diseases, Cancer genomics

## Abstract

Clonal hematopoiesis (CH) can be caused by either single gene mutations (eg point mutations in *JAK2* causing CHIP) or mosaic chromosomal alterations (e.g., loss of heterozygosity at chromosome 9p). CH is associated with a significantly increased risk of hematologic malignancies. However, the absolute rate of transformation on an annualized basis is low. Improved prognostication of transformation risk is urgently needed for routine clinical practice. We hypothesized that the co-occurrence of CHIP and mCAs at the same locus (e.g., transforming a heterozygous *JAK2* CHIP mutation into a homozygous mutation through concomitant loss of heterozygosity at chromosome 9) might have important prognostic implications for malignancy transformation risk. We tested this hypothesis using our discovery cohort, the UK Biobank (*n* = 451,180), and subsequently validated it in the BioVU cohort (*n* = 91,335). We find that individuals with a concurrent somatic mutation and mCA were at significantly increased risk of hematologic malignancy (for example, In BioVU cohort incidence of hematologic malignancies is higher in individuals with co-occurring *JAK2* V617F and 9p CN-LOH; HR = 54.76, 95% CI = 33.92–88.41, *P* < 0.001 vs. *JAK2* V617F alone; HR = 44.05, 95% CI = 35.06–55.35, *P* < 0.001). Currently, the ‘zygosity’ of the CHIP mutation is not routinely reported in clinical assays or considered in prognosticating CHIP transformation risk. Based on these observations, we propose that clinical reports should include ‘zygosity’ status of CHIP mutations and that future prognostication systems should take mutation ‘zygosity’ into account.

## Introduction

Clonal hematopoiesis (CH) is an age-related acquisition of mutations that improve cellular fitness and result in the selective expansion of mutated hematopoietic stem cells. CH is the pre-malignant lesion that precedes hematologic malignancy, including both mutations in myeloid driver genes (CHIP) [1–3] and somatic copy number variants (mosaic chromosomal alterations, mCAs) [4–6]. However, most individuals with CHIP do not progress to hematologic malignancy. Several risk factors contributing to the risk of progression include the CHIP driver gene, the clonal fraction of the mutation, and the clonal complexity. Additional factors, like the specific mutated residue within a given gene, are also posited to contribute to progression risk. More recently, the development of a CH risk score (CHRS) to predict an individual risk of developing blood cancer, which includes a patient’s age, the type and number of genetic mutations present in blood cells, the fraction of cells in the blood with the mutation; low blood counts; and factors related to red blood cell volume [7].

We hypothesized that whether an individual was heterozygous (e.g., had one copy of the driver mutation) or homozygous (e.g., had two copies of the driver mutation) is a significant risk factor in the progression of CH to malignancy. To test this hypothesis, we initially queried all the canonical CHIP mutations (74 mutations) in a large dataset (UK Biobank). However, we were limited in our analysis given the rarity of the combinations (CHIP and mCAs). So, subsequently, we focused on two of the most common CHIP mutations, *DNMT3A* R882 and *JAK2* V617F, to avoid heterogeneity that may exist from different alleles conferring different disease risks. We characterized the co-occurrence of these mutations with mCAs and how this co-occurrence is associated with progression risk.

## Methods

### UK Biobank

UK Biobank (UKB) was used as a discovery cohort. We identified CHIP in 451,180 individuals in the UK Biobank with available exome data and without hematologic cancers diagnosed prior to or within six months of blood draw. mCA detection in the UK Biobank was previously described [8]. CHIP variants meeting previously defined criteria [9] were filtered using established filtering criteria [10]. In summary, the 74 canonical CHIP genes were scanned for putative CHIP mutations using the *Mutect2* somatic variant caller [9]. Variants present in a pre-established list of candidate CHIP variants with total sequencing depth (DP) ≥ 20, alternate allele read depth count (minAD) ≥ 5, and present in reads in both sequencing directions (i.e., F1R2 ≥ 1 and F2R1 ≥ 1) were included in the preliminary dataset. We then implemented population-based filtering parameters to remove suspected false positive variants. These filtering parameters are described in detail in the Methods paper [10].

### BioVU

BioVU was used as a replication cohort. Data on 91,335 patients from the BioVU repository [11], a Vanderbilt University Medical Center biobank with linked de-identified electronic health records (EHRs), spanning 2006 to 2021, were included in the association analyses (Supplemental Fig. S1). In the BioVU cohort, individuals with a diagnosis of hematologic malignancy (HM) prior to or within six months of the blood draw were excluded (n = 980), while those who went on to develop HM post-blood draw were included in the analyses. Of these 91,335 individuals in the cohort, 1615 individuals were reported to have developed HM subsequently: 947 myeloid and 668 lymphoid (Supplemental Fig. S1). ICD-9 and ICD-10 codes were used to categorize the phenotypes. Cases were defined as individuals with three distinct HM ICD codes on different days. The patients without any ICD codes of HM were selected as controls. Patients with only one or two ICD codes were excluded from the analysis.

### mCA calls in BioVU

Peripheral blood-derived DNA of 91,335 subjects from BioVU were genotyped on the Illumina Expanded Multi-Ethnic Genotyping Array (MEGA^EX^) platform, which contains more than 2 million single-nucleotide polymorphisms (SNPs). Detection of mCAs in the BioVU was performed starting from raw IDAT files from the Illumina MEGA^EX^, as previously described [8]. We defined mosaic chromosomal alteration (mCA) by focusing solely on the autosomal mCA calls and excluding any loss of X and Y events. The mCA calls that we included were copy-neutral loss of heterozygosity (CN-LOH), loss of chromosomal regions, gains of chromosomal regions, and mCAs of unknown copy change.

### CHIP calls in BioVU

SNV/indel mutation calls (*DNMT3A* R882/*JAK2* V617F) were ascertained among ~100 K research participants with MEGA^EX^ genotyping, which was completed as part of an institutional characterization of participants in the BioVU biobank. In its design, MEGA^EX^ includes specific probe sets that interrogate *JAK2* V617F and *DNMT3A* R882C and R882H. Each probe set includes one probe that specifically hybridizes to the mutant allele and one that hybridizes to the wild-type (WT) allele, generating raw intensity values for both alternate (X) and reference (Y) alleles. Individuals were designated as having a mutation if their normalized alternate allele fraction was 6 standard deviations greater than the population mean, as has been done previously [12]. For validation of MEGA^EX^-based CHIP calls, DNA samples stored in BioVU from 477 participants with MEGA^EX^ genotyping were selected for gold-standard Next Generation Sequencing(NGS). Sequencing was performed to >500× coverage at the Vanderbilt sequencing core using a custom-capture panel consisting of 24 commonly mutated CHIP genes. There was a very high concordance between MEGA^EX^ -identified CHIP for *DNMT3A* R882C and *JAK2* V617F (*R*^2^ > 0.9), but less so for *DNMT3A* R882H. Consequently, BioVU participants were categorized as *DNMT3A* R882 positive if they were positive for *DNMT3A* R882H via NGS or *DNMT3A* R882C on MEGA^EX^ prediction.

### Risk factors for CH in BioVU

To identify risk factors for CH, we examined associations between various genetic alterations and subjects’ baseline characteristics in BioVU (age, gender, race, BMI, history of smoking, diagnosis of hypertension, diabetes, and hyperlipidemia) (Supplemental Table S1). Females, white race, non-smokers, and BMI ≦ 30 were reference groups. First, we performed a multivariate logistic regression for age, gender, and race. Thereafter, to control the effect of age, gender, and race on CH, we performed logistic regressions for other characteristics adjusted age, gender, and race to identify significant risk factors (*P* < 0.05).

### Effect of CH on blood cell counts

To elucidate the effects of genetic alterations on blood cell counts, we examined correlations between genetic alterations and blood cell counts (RDW, PLT, HB, WBC, MCV). After grouping subjects by the definition of abnormalities in blood counts, logistic regressions were performed. To correct for confounding effects, all regressions were performed with multivariate models, including baseline characteristics as covariates, in comparison with subjects without genetic alterations in CH.

### Statistical analyses

The relationship between independent variables and disease progression was assessed using Cox proportional hazards model. All statistical analyses were performed using the R package “survival” v.3.3-1.

## Results

**Fig. 1:**
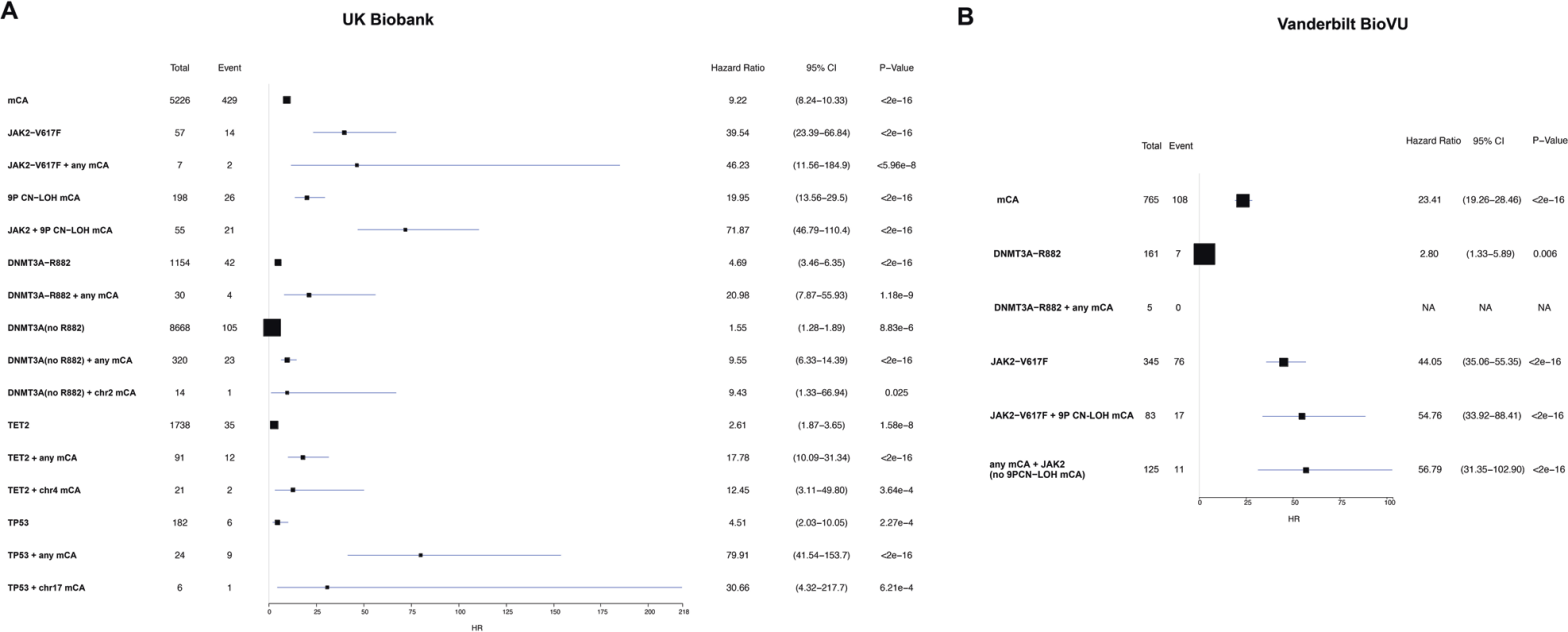
CH-alterations and risk of hematologic malignancies. **A** Increased risk of hematologic malignancy in the UK Biobank cohort with or without mCAs, *DNMT3A/JAK2/TET2/TP53*. **B** Increased risk of hematologic malignancy in the BioVU cohort with or without mCAs, *DNMT3A/JAK2*, and both.

We analyzed 451,180 individuals in the UKB, we identified autosomal mCAs in 5745 (1.3%) individuals in the UKB cohort. The most frequently detected mCAs in this cohort were involving chromosome 1 (*n* = 809), chromosome 11 (*n* = 742), chromosome 22 (*n* = 734), and chromosome 14 (*n* = 628). CHIP mutation calls were identified in 15,304 (3.4%) individuals. The most commonly mutated CHIP gene was *DNMT3A* (*n* = 8988), followed by *TET2* (*n* = 1829) and *ASXL1* (*n* = 1545). Specific alleles of *DNMT3A* R882 were identified in 1152 (0.3%), and *JAK2* V617F was identified in 112 (0.02%) subjects. The presence of mCAs (HR = 9.22, 95% CI = 8.24–10.33, *p* < 0.001), *DNMT3A* R882 (HR = 4.69, 95% CI = 3.46–6.35, *p* < 0.001), *JAK2* V617F (HR = 39.54, 95% CI = 23.39 = 66.84, *p* < 0.001), *TET2* (HR = 2.61, 95% CI = 1.87-3.65, *P* < 0.001), and *TP53* (HR = 4.51, 95% CI = 2.03-10.05) were all associated with a higher incidence of HM in the UKB cohort (Fig. 1A). The co-occurrence of any mCAs with *DNMT3A* R882 mutations was not elevated above the risk of mCAs alone (HR = 20.98, 95% CI = 7.87–55.93, *P* < 0.001) as the point estimate for mCAs alone fell within the 95% confidence interval. Individuals with *JAK2* V617F, *TET2*, and *TP53* CHIP mutations in combination with a 9 P CN-LOH, chromosome 4 mCA, and chromosome 17 mCA, respectively, transformed the heterozygous mutation to a homozygous mutation and were at significantly elevated risk of HM above and beyond the risk of heterozygous *JAK2* V617F, *TET2*, and *TP53* (e.g., individuals without a 9 P CN-LOH mutation, chromosome 4 mCA, and chromosome 17 mCA) (Fig. 1A). In the UKB, of the 26 individuals with *JAK2* V617F and concurrent autosomal mCAs who developed incident hematologic cancers, there were 10 cases of polycythemia vera, 4 cases of myeloproliferative neoplasm NOS, 4 cases of myelofibrosis, 3 cases of essential thrombocythemia, 2 cases of AML, and 1 case of each of the following: APML, CML, and MDS.

**Fig. 2:**
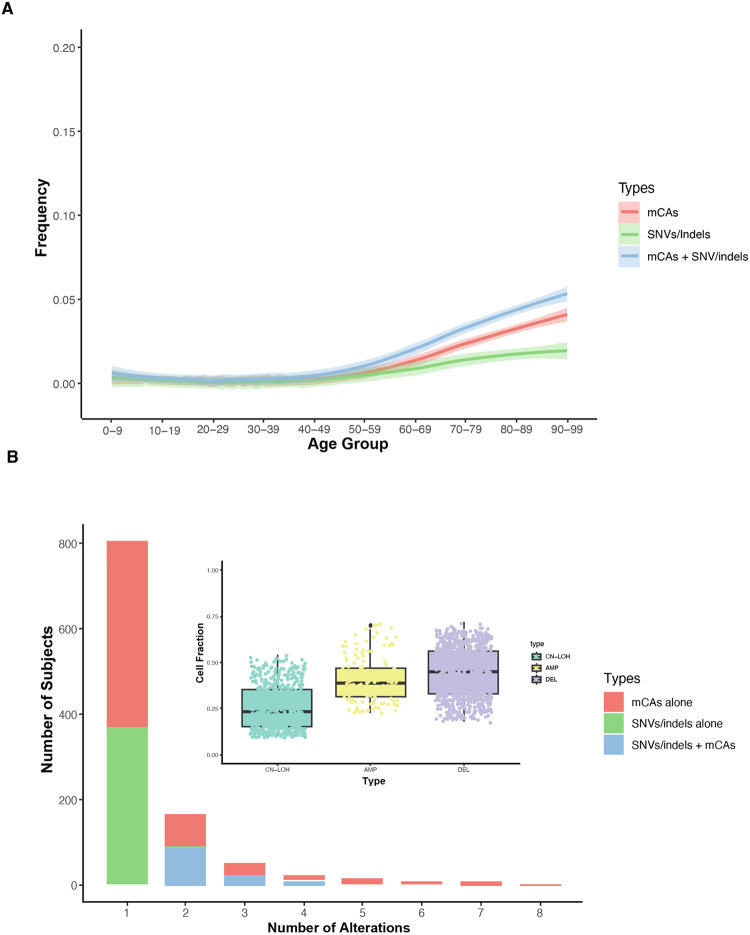
Landscape of mCAs and SNVs/indels. **A** The prevalence of mCAs, SNV/indels and combined increases with age in the BioVU cohort; shaded bands indicate the estimated 95% CI. **B** Distribution of the number of genetic alterations in each subject. **C** Distribution of detected mCAs with cell fractions.

To replicate these initial findings from the UKB, we tested our hypotheses in the BioVU cohort. We identified autosomal mCAs in 765 (0.8%) individuals in the BioVU cohort by applying the MoChA algorithm to the genotyping arrays [8]. Most frequent mCAs detected in this cohort include 9p CN-LOH (*n* = 105, 6.9%), del [5q] (*n* = 99, 6.5%), del [20q] (*n* = 98, 6.4%), del [7q] (*n* = 94, 6.2%), and del [13q] (*n* = 67, 4.4%). CHIP mutation calls (*DNMT3A* R882 and *JAK2* V617F) based on the Illumina MEGA^EX^ array intensity data were identified in 503 (0.6%) individuals (Fig. 2B). Of these, *DNMT3A* R882 were identified in 161 (0.2%), and *JAK2* V617F were identified in 345 (0.4%) subjects. Salient features comparing the UK biobank cohort with the BioVU cohort are described in Supplemental Table S2. As shown previously [1–3, 13, 14], the presence of mCAs and *DNMT3A* R882/*JAK2* V617F are strongly age-related, with an increased frequency in the elderly (Fig. 2A).

In the BioVU, combined mCAs and *DNMT3A* R882/*JAK2* V617F genetic alterations were reported in 130 (0.1%) individuals. Similar to the UK Biobank, co-occurring *JAK2* V617F and 9p CN-LOH (*n* = 100, OR = 5693 *q* < 0.001) are consistent with the well-known mechanisms of bi-allelic alterations in specific genes driving the pathogenesis of hematologic malignancies.

Next, we sought to replicate our primary findings from the UK Biobank and establish whether the presence of co-occurring mCA and *JAK2*/*DNMT3A* impact the risk of subsequent development of hematologic malignancies (HM). In the BioVU, mCAs were associated with a higher incidence of HM (hazard ratio (HR) = 23.41, 95% confidence interval (CI) 19.26–28.46, *P* < 0.001). *JAK2* V617F was also associated with a higher incidence of HM (HR 44.05, 95% CI 35.06–55.35, *P* < 0.001), but *DNMT3A* R882 was not. The co-occurrence of *DNMT3A* R882 with any mCA did not elevate the risk of HM. Conversely, individuals with a concurrent *JAK2* V617F and 9p copy neutral loss-of-heterozygosity event, which transforms the *JAK2* V617F mutation from a heterozygous mutation to a homozygous mutation, were at significantly increased risk of HM (HR = 54.76, 95% CI = 33.92-88.41, *P* < 0.001) (Fig. 1B). In the BioVU cohort, 17 individuals with JAK2 V617F and 9p CN-LOH developed hematological cancers. There were 10 cases of myeloproliferative neoplasm, 4 cases of MDS, 2 cases of CML, and 1 case of AML.

Although the demographic factors, co-morbidities, and lifestyle factors impacting *DNMT3A* R882, *JAK2* V617F, and mCAs have been previously explored in the UK Biobank [15], this is the first report of CH in BioVU. Therefore, we sought to elucidate these relationships in the BioVU cohort. Male gender is significantly associated independently with *DNMT3A*/*JAK2* and mCAs. The effect of the association increased if we combined *DNMT3A*/*JAK2* and mCAs (Supplemental Figure S2A). Furthermore, we explore the landscape of blood count characteristics and its impact on various types of CH. Individuals with mCAs are associated with lower WBC, platelet count, and any blood count abnormality (Supplemental Fig. S2B). The presence of both individual mCAs and SNV/indels was significantly correlated with blood count abnormalities. For example, a high platelet count had a higher frequency of *JAK2* V617F and 9p CN-LOH (OR = 28.47, *P* = 2.05e−38) (Supplemental Fig. S2B).

## Discussion

Here, we show that CHIP mutation zygosity is an important contributor to risk stratification of CH patients in two large datasets and should be reported in clinical reports of molecular tests noting CHIP in addition to other features with prognostic significance, like the gene mutation and the clonal fraction. In contrast to prior work [15, 16], which considered the co-occurrence of mCAs and SNVs as a class, we were able to demonstrate the effect of zygosity at the resolution of single CH driver mutations, minimizing heterogeneity introduced by different CH driver genes and mutations. We find that *JAK2* V617F homozygotes have increased risk above heterozygotes in two datasets. Conversely, simply having any mCA and a *DNMT3A* R882 mutation does not increase risk above any mCA alone. Our work also differs from prior efforts in that the BioVU cohort includes all age groups, and previous publications looking into the landscape of both mCAs and SNV/indels were restricted to the elderly population [16] or biased towards the cancer population [17].

**Fig. 3:**
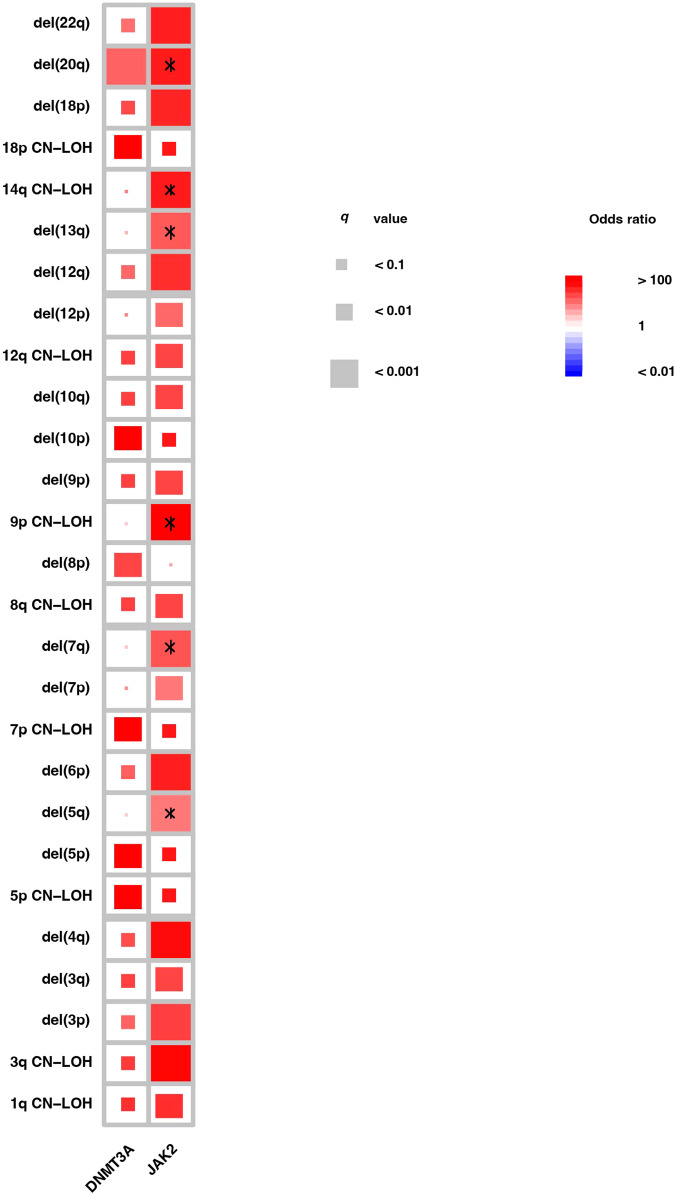
Correlation between selective mCAs and SNV/indels (*DNMT3A*/*JAK2*) in the BioVU cohort.

Interestingly, we find that the model for mCAs gains a selective advantage, leading to clonal expansion mediated through bi-allelic alterations, which is not uniformly true. For example, 9pCN-LOH significantly co-occurred with *JAK2* V617F (*n* = 100, OR = 5693 *q* < 0.001); however, *DNMT3A* R882 did not have a similar increase in chromosome 2 LOH events. We also report the increased co-occurrence of *JAK2* V617F (but not *DNMT3A*) and 14q CN-LOH (Fig. 3 and Supplemental Fig. S5). One potential explanation of these findings is a recent description of the fitness advantage of numerous genetic alterations in CH being mediated through TCL1A activation [18, 19]. Interestingly, TCL1A is located on chromosome 14q32, which has been associated with an inherited predisposition to the development of myeloid malignancies [20]. These results further elucidate that various mCAs gain selective advantage through complex patterns of co-mutations to drive the development of CH and, eventually, the predisposition of hematologic malignancies.

The current study has several limitations. While both CHIP mutations and mCAs were observed, their co-occurrence was not evaluated using multivariate analyses. This decision was based on the insufficient number of co-occurrence events in conjunction with other predictors of hematologic malignancies, as delineated in the CHRS risk model. Another potential limitation is the focus on the two most common CHIP driver mutations in our BioVU replication cohort. Although this enabled single variant level resolution and minimized heterogeneity introduced by other genes and variants, it limits the generalizability of our findings to other mutations and genes. In future studies, we aim to expand our current analyses to include additional recurrently mutated genes known to be drivers in CH through expanded CHIP sequencing of the BioVU cohort.

## Conclusion

Since CH was first described a decade ago, understanding which patients are at the highest risk for the development of hematologic malignancies has been a critical goal. To that end, our current study using two large cohorts demonstrates that comprehensive profiling of both mCAs and SNV/indels enables the identification of the ‘zygosity’ status of the mutation, which has important prognostic significance and should be incorporated into risk stratification systems and clinical reports.

### Supplementary information

Supplemental material

S1

S2

S3

S4

S5

S1

S2

## Data Availability

UK Biobank data is available upon application through a procedure described at https://www.ukbiobank.ac.uk/enable-your-research. Vanderbilt BioVU data is available through an application to the Vanderbilt Institute for Clinical and Translational Research BioVU Review Committee.

## Code availability

The code to call mosaic chromosomal alterations with MoChA is available here https://github.com/freeseek/mocha.
