# Supplementary material for: Driver mutation zygosity is a critical factor in predicting clonal hematopoiesis transformation risk
Source: Blood Cancer J. 2024 Jan 15;14(1):6. doi: 10.1038/s41408-023-00974-9 (PMC10789770; doi:10.1038/s41408-023-00974-9)
Supplement: Supplementary file 1 — Supplemental material [file 41408_2023_974_MOESM1_ESM.docx]

**Supplemental information**

**Driver Mutation Zygosity is a Critical Factor in Predicting Clonal Hematopoiesis Transformation Risk**

Ashwin Kishtagari^1,2 #^, M.A. Wasay Khan^3 #^, Yajing Li^4 #^, Caitlyn Vlasschaert^5^, Naimisha Marneni^1,2^, Alexander J. Silver^6^, Kelly von Beck^7^, Travis Spaulding^1,2^, Shannon Stockton^1,2^, Christina Snider^8^, Andrew Sochacki^1,2^, Dixon Dorand^1,2^, Taralynn M. Mack^3^, P Brent Ferrell Jr^1,2^, Yaomin Xu^4,9,10^, Cosmin A. Bejan^10^, Michael R. Savona^1,2,6,11^ **, Alexander G. Bick^3,6^ **

^1^ Division of Hematology/Oncology – Vanderbilt University School of Medicine, Nashville, TN, USA.

^2^ Vanderbilt-Ingram Cancer Center – Vanderbilt University School of Medicine, Nashville, TN, USA.

^3^ Division of Genetic Medicine – Vanderbilt University Medical Center, Nashville, TN, USA.

^4^ Department of Biostatistics, Vanderbilt University School of Medicine, Nashville, TN

^5^ Department of Medicine – Queen’s University, Kingston, ON, Canada.

^6^ Program in Cancer Biology, Vanderbilt University School of Medicine, Nashville, TN, USA.

^7^ Vanderbilt University School of Medicine, Nashville, TN, USA.

^8^ Department of Medicine, Vanderbilt University Medical Center, Nashville, TN

^9^ Center for Quantitative Sciences, Vanderbilt University School of Medicine, Nashville, TN

^10^ Department of Biomedical Informatics, Vanderbilt University School of Medicine, Nashville, TN

^11^ Center for Immunobiology – Vanderbilt University School of Medicine, Nashville, TN, USA.

# Contents:

**Supplemental Figures and Tables**

# A) Supplemental figures

# Figure S1: CONSORT diagram of the BioVU cohort selection.

# Figure S2: A. Association of mCAs, SNV/indels, and combined with hyperlipidemia, diabetes, hypertension, smoking status, body mass index (BMI)>30, race, age, and gender. B. Enrichment of genetic alterations in cases with abnormalities in blood counts.

# Figure S3: Individual mCAs and risk of myeloid and lymphoid neoplasms in the BioVU cohort

# Figure S4: Increased risk of hematologic malignancy in the UK Biobank cohort with or without mCAs, *DNMT3A/JAK2/TET2/TP53,* stratified by variant allele frequency (VAF).

# Figure S5: Correlation between individual mCAs and SNV/indels (*DNMT3A*/*JAK2*) in the BioVU cohort.

# B) Supplemental tables

- **Table S1:** Baseline demographics and comorbidities of the BioVU cohort. HM (+/-) Subjects with/without the event of hematologic malignancy during follow-up periods. OR= Odds Ratio, CI=Confidence Interval
- **Table S2:** Salient features comparing the UK biobank and the BioVU cohorts.
