# Supplementary figures and images for: Driver mutation zygosity is a critical factor in predicting clonal hematopoiesis transformation risk
Source: Blood Cancer J. 2024 Jan 15;14(1):6. doi: 10.1038/s41408-023-00974-9 (PMC10789770; doi:10.1038/s41408-023-00974-9)

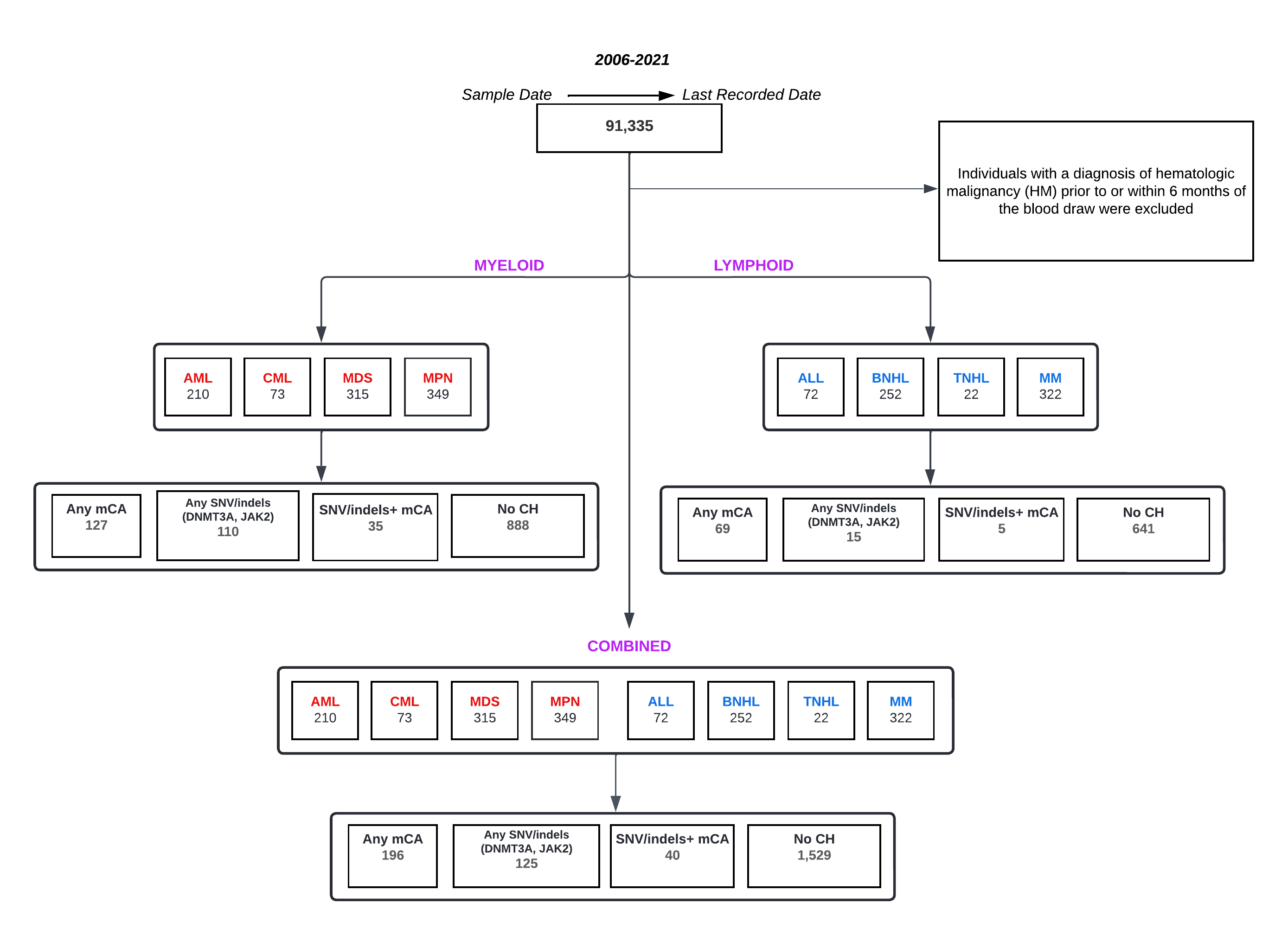

Supplement: Supplementary file 2 — S1 [file 41408_2023_974_MOESM2_ESM.png]

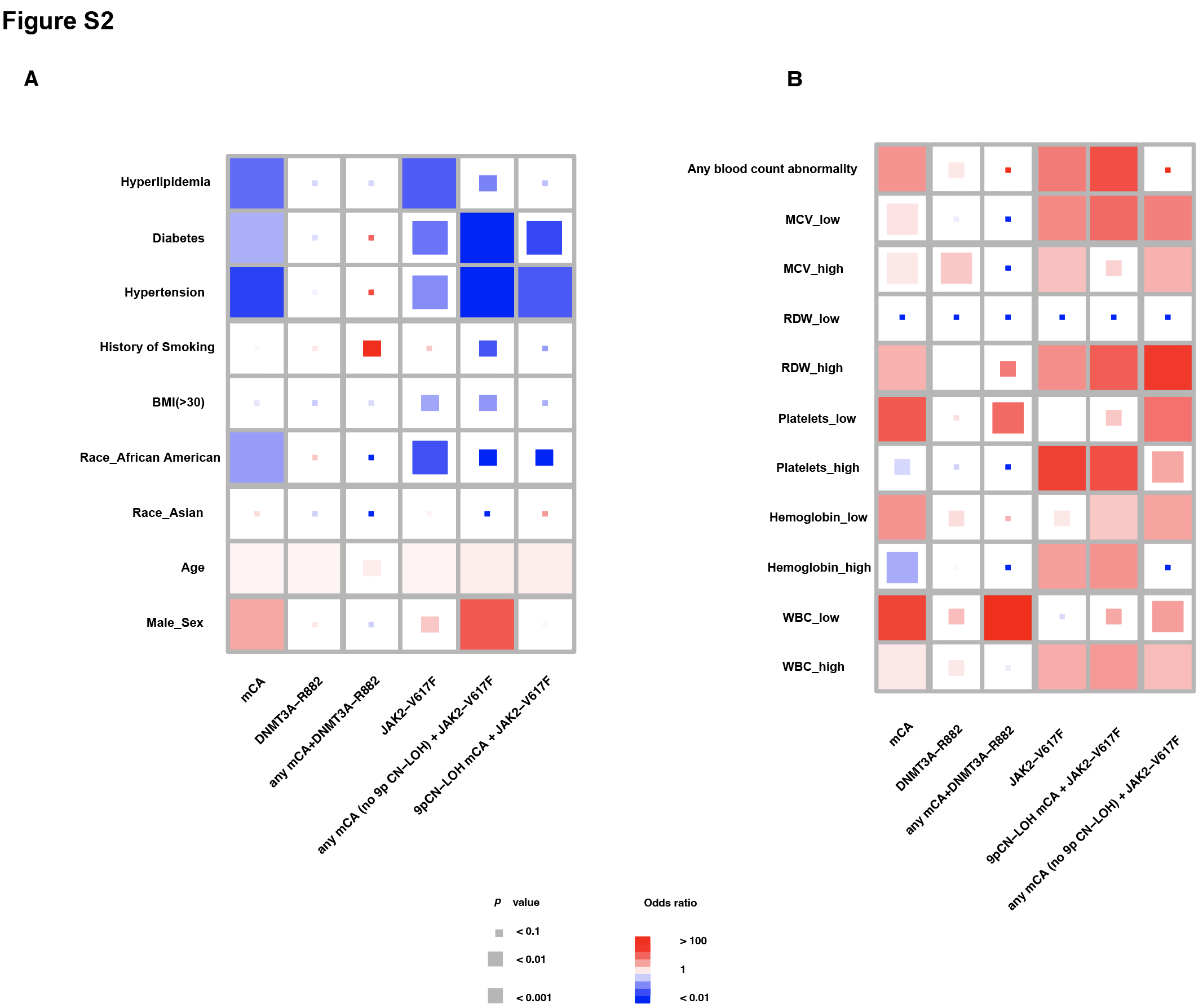

Supplement: Supplementary file 3 — S2 [file 41408_2023_974_MOESM3_ESM.png]

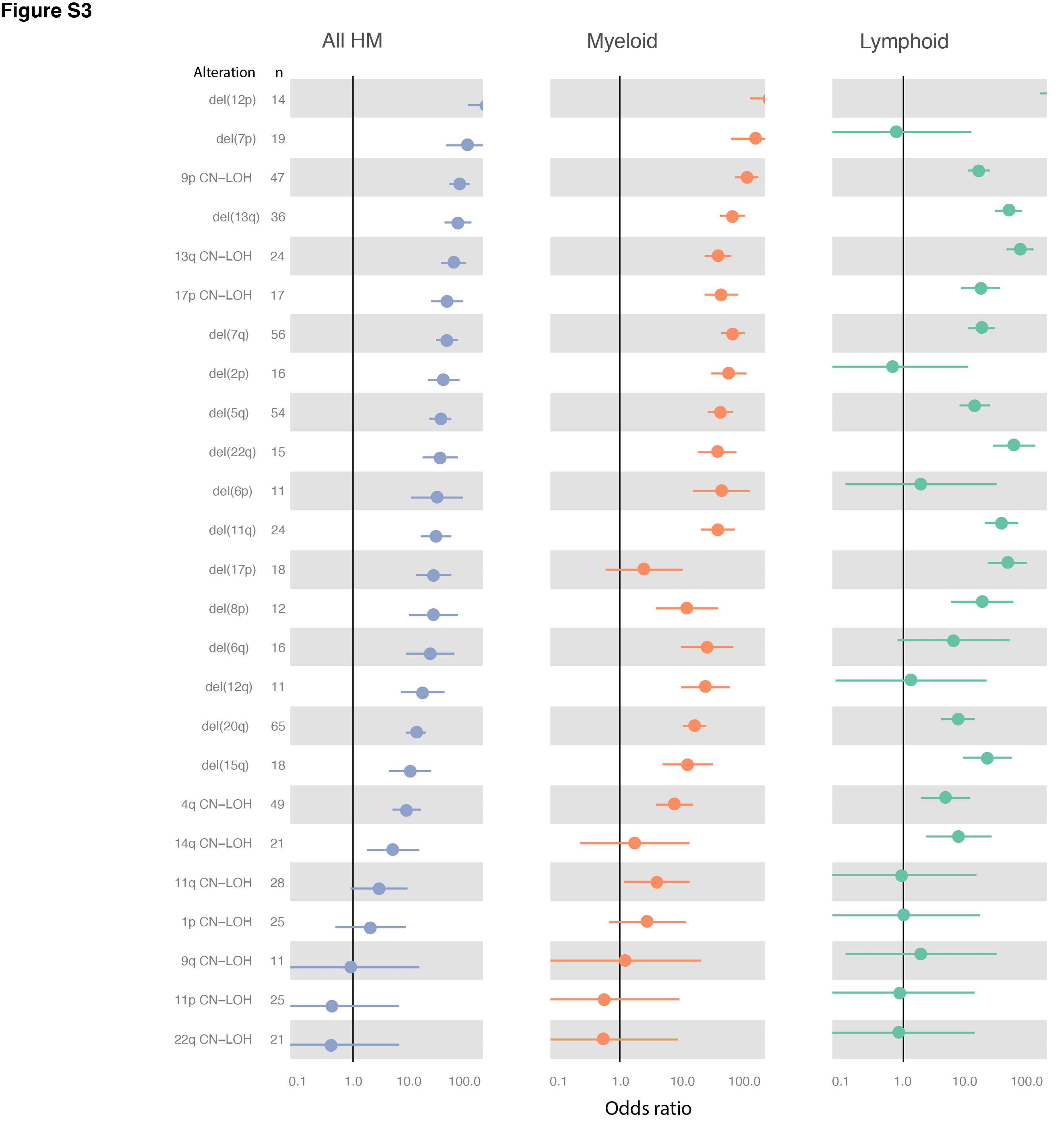

Supplement: Supplementary file 4 — S3 [file 41408_2023_974_MOESM4_ESM.jpg]

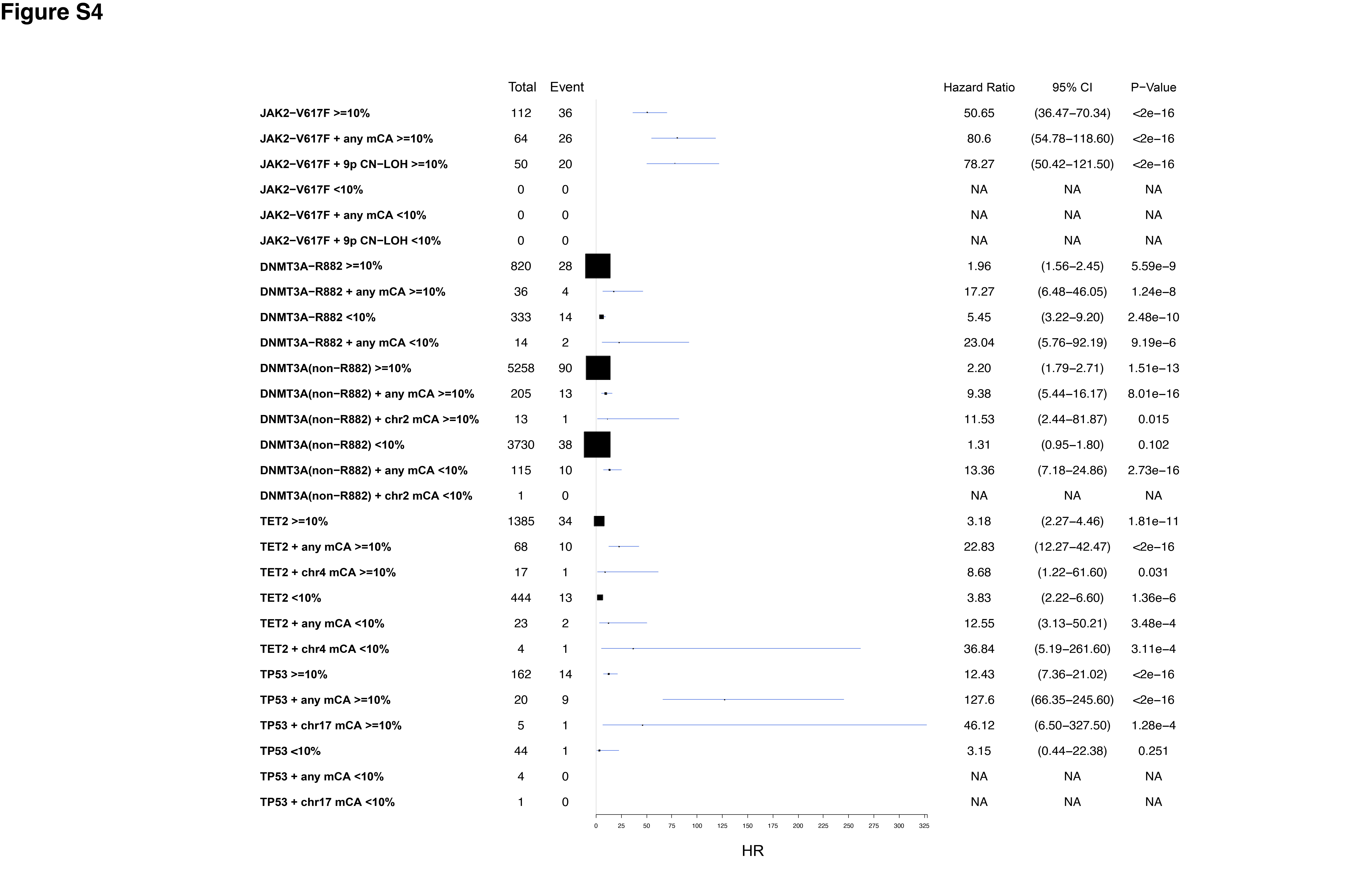

Supplement: Supplementary file 5 — S4 [file 41408_2023_974_MOESM5_ESM.jpg]

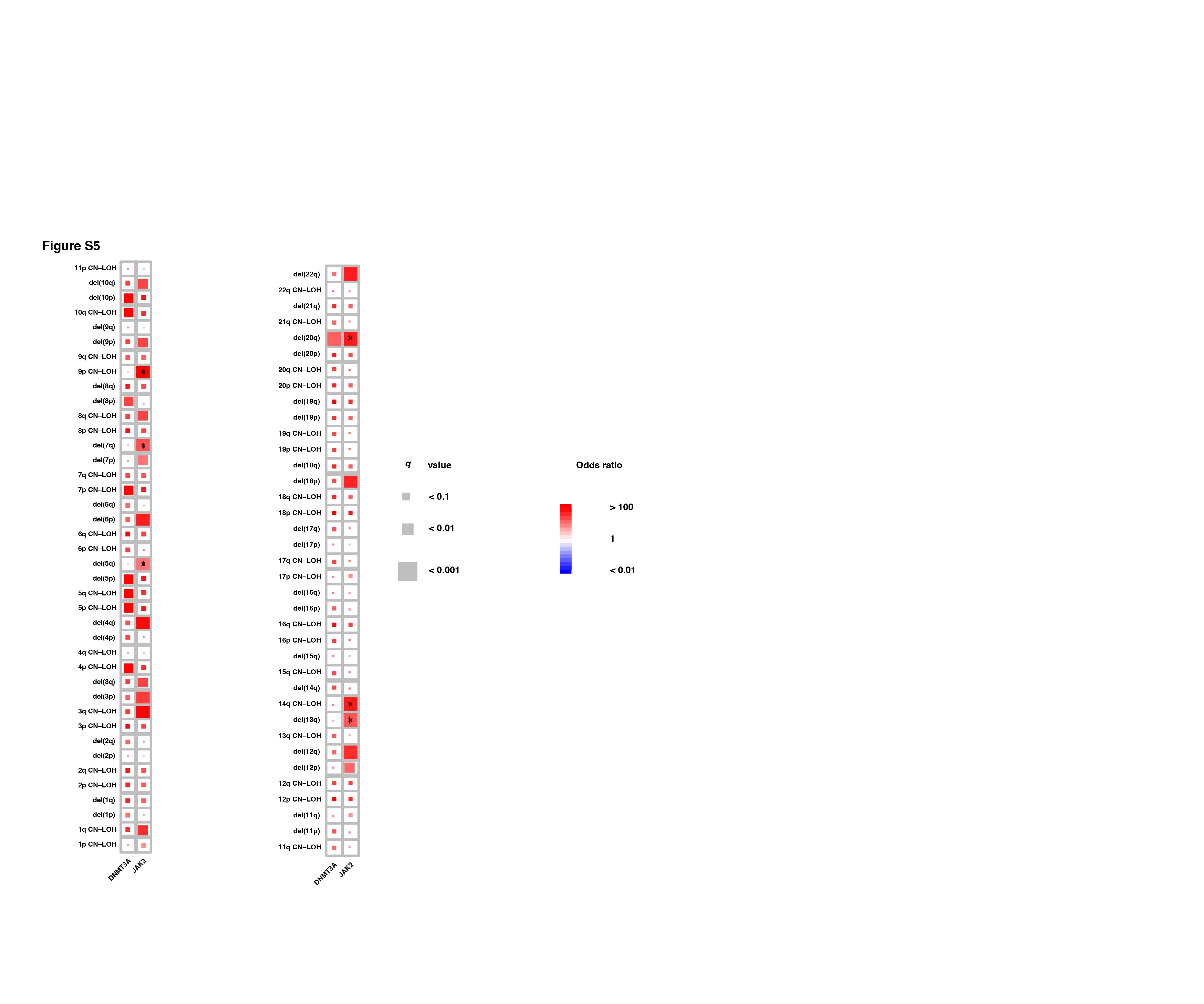

Supplement: Supplementary file 6 — S5 [file 41408_2023_974_MOESM6_ESM.jpg]
